# Soluble TREM‐1, as a new ligand for the membrane receptor Robo2, promotes hepatic stellate cells activation and liver fibrosis

**DOI:** 10.1111/jcmm.17033

**Published:** 2021-11-09

**Authors:** Ting Liu, Shujia Chen, Xiaoli Xie, Hongqun Liu, Yongjuan Wang, Shengbin Qi, Linping Shi, Xue Zhou, Jiuna Zhang, Shuling Wang, Yijun Wang, Shengxiong Chen, Shiying Dou, Xiaoyu Jiang, Ruolin Cui, Huiqing Jiang

**Affiliations:** ^1^ Department of Gastroenterology The Second Hospital of Hebei Medical University Hebei Key Laboratory of Gastroenterology Hebei Institute of Gastroenterology Hebei Clinical Research Center for Digestive Diseases Shijiazhuang Hebei China; ^2^ Department of Gastroenterology Shijiazhuang People’s Hospital Shijiazhuang Hebei China; ^3^ Department of General Surgery Shijiazhuang People’s Hospital Shijiazhuang Hebei China; ^4^ Department of Gastroenterology Hebei General Hospital Shijiazhuang Hebei China; ^5^ Department of Hepatobiliary Surgery The Second Hospital of Hebei Medical University Shijiazhuang Hebei China; ^6^ Department of infectious diseases The Second Hospital of Hebei Medical University Shijiazhuang Hebei China

**Keywords:** hepatic stellate cell, ligand, liver fibrosis, pro‐inflammatory factor, receptor, roundabout guidance receptor 2 (Robo2), soluble triggering receptor expressed on myeloid cells‐1 (sTREM‐1)

## Abstract

Triggering receptor expressed on myeloid cells‐1 (TREM‐1) exists in two forms: a transmembrane form and a soluble form (sTREM‐1). The levels of sTREM‐1 are elevated in supernatants of activated HSCs. However, the role of sTREM‐1 in HSC activation and liver fibrosis remains undefined. Previous studies have primarily focused on the transmembrane form of TREM‐1; we innovatively observed the function of sTREM‐1 as a ligand in liver fibrosis and screened its receptor. Here, recombinant sTREM‐1 was used as a stimulator which induced HSC activation and further aggravated liver fibrosis. Then, screening for sTREM‐1 interacting membrane receptors was performed using pull‐down assay followed by mass spectrometry, and the membrane receptor roundabout guidance receptor 2 (Robo2) was identified as a candidate receptor for sTREM‐1. The interaction between sTREM‐1 and Robo2 was verified by pull‐down and immunofluorescence. The role of Robo2 on sTREM‐1‐induced HSC activation and its downstream signal pathways was assessed by knockdown of Robo2 in LX‐2 cells. Furthermore, HSC‐specific knockdown of Robo2 was achieved in a mouse model of liver fibrosis by using a recombinant adeno‐associated virus (AAV) vector to confirm the role of the receptor, and we proved that Robo2 knockdown inhibited the activation of HSC and liver fibrosis, which also led to the inactivation of Smad2/3 and PI3K/Akt pathways in sTREM‐1‐induced HSC activation and liver fibrosis. In conclusion, sTREM‐1 acts as a new ligand of Robo2; the binding of sTREM‐1 to Robo2 initiates the activation of the downstream Smad2/3 and PI3K/Akt signalling pathways, thereby promoting HSC activation and liver fibrosis.

## 1 INTRODUCTION

The continuous progress of liver fibrosis is the sticking point to the development of chronic liver disease to cirrhosis, but there is currently no effective anti‐fibrosis treatment.^1^, ^2^ Revealing the mechanism of liver fibrogenesis is fundamental for exploring the effective treatment.^3^ Inflammation is an important initiating and driving factor of liver fibrosis. During the liver fibrosis, various inflammatory factors are released and related inflammatory pathways are activated, resulting in the activation of hepatic stellate cells (HSCs) and collagen deposition.^4^ The activation of HSC is the core event of liver fibrosis.^5^ The inflammatory response is involved in the entire process of HSC activation, phenotypic transformation and collagen secretion.^6^ Therefore, inflammatory‐related cytokines and the inflammatory response mediated by them play an important role in the activation of HSC.

Triggering receptor expressed on myeloid cells‐1 (TREM‐1) belongs to the immunoglobulin (Ig) superfamily. Its basic structure includes the transmembrane domain, V‐type immunoglobulin‐like extracellular domain and cytoplasmic domain. It distributes on the surface of monocytes, neutrophils, intestinal epithelial cells and HSCs.^7^ There are two main forms of TREM‐1 in the organism: one is a structurally complete membrane receptor that exists on the cell membrane; the other is a free form that lacks a transmembrane domain and is known as soluble triggering receptor expressed on myeloid cells‐1 (sTREM‐1), which is a secreted protein that can be detected in serum.^8^ Heretofore, studies mainly used TREM‐1 as a receptor, but we innovatively utilized sTREM‐1 as a ligand in liver fibrosis to find the receptor that interacts with it.

Previous studies have found that the serum levels of sTREM‐1 in patients with various inflammatory diseases, such as bacteraemia, acute pancreatitis and rheumatoid arthritis, are significantly increased and are associated with poor prognosis.^9^, ^10^, ^11^, ^12^, ^13^, ^14^ In recent years, studies have used sTREM‐1 as a serological marker to assess the severity and prognosis of diseases.^10^, ^11^, ^12^, ^13^, ^14^, ^15^ Therefore, sTREM‐1 is a cytokine closely related to inflammation. sTREM‐1 is significantly increased in the serum of patients with hepatocellular carcinoma (HCC). Interestingly, co‐culture of HSC with HCC significantly increases the secretion of sTREM‐1 from activated HSCs.^16^ However, Liao's study only focused on the expression of sTREM‐1 and did not explore its role in the liver disease. In addition, sTREM‐1 is a protein lacking the transmembrane domain and cannot pass through the cell membrane.^17^, ^18^ Therefore, sTREM‐1 only functions as an extracellular signalling molecule that acts on a receptor on the cell membrane.^19^, ^20^

The present research systematically studied the cell membrane receptor bound by the sTREM‐1 and the downstream signalling pathways, clarified the role and mechanism of sTREM‐1 in liver fibrosis, provided new mechanisms of liver fibrosis and supplied new intervention target for the treatment.

## 2 MATERIALS AND METHODS

### 2.1 Animal models

#### 2.1.1 Set 1. Liver fibrosis model

Six‐week‐old male C57BL/6J mice were purchased from Beijing Vital River Laboratory Animal Technology Co., Ltd. After one week of acclimation, mice were randomly divided into the Oil (Shanghai Aladdin Biochemical Technology Co., Ltd., Shanghai, China; Catalog number: O108686; *n* = 6), Oil+sTREM‐1 (1 μg g^−1^, Sino Biological Inc., Beijing, China, *n* = 6), carbon tetrachloride (CCl_4_, Shanghai Aladdin Biochemical Technology Co., Ltd.; Catalog number: C112045; *n* = 6) and CCl_4_+sTREM‐1 groups. The CCl_4_ group received intraperitoneal injections of CCl_4_ (5 μl g^−1^, mixed with olive oil, 10% CCl_4_) two times per week for a total of six weeks, and the Oil group was injected with only olive oil for the same dose and time. The mice in Oil+sTREM‐1 and CCl_4_+sTREM‐1 group were injected with recombinant mouse sTREM‐1 (1 μg g^−1^) through the tail vein at the same time as CCl_4_ treatment on the day after CCl_4_ injection, and the Oil and CCl_4_ groups were injected with phosphate‐buffered saline (PBS, Solarbio Science & Technology Co., Ltd., Beijing, China; Catalog number: P1020) through the tail vein for the same dosage and period.

#### 2.1.2 Set 2. sTREM‐1 testing

Six‐week‐old male C57BL/6J mice were divided into 2 groups; sTREM‐1 mice (*n* = 6) were injected with recombinant mouse sTREM‐1 intraperitoneally (1 μg g^−1^, twice per week for sixteen weeks) to observe whether it induces liver fibrosis in mice. The mice in control group (*n* = 6) were injected with PBS intraperitoneally for the same volume and period.

#### 2.1.3 Set 3. Roundabout guidance receptor 2 (Robo2) knockdown by recombinant adeno‐associated viruses (AAV) administration

Three small interfering ribonucleic acids (siRNAs) targeting different targets of Robo2 (Shanghai GenePharma Co., Ltd., Shanghai, China) were designed and screened out the valid sequence for knocking down Robo2 in mouse colon cancer cell line CT26. The cells were cultured in RPMI 1640 medium (Gibco BRL, Grand Island, NY, USA; Catalog number: 61870036) supplemented with 10% foetal bovine serum (FBS, HyClone, South Logan, UT, USA; Catalog number: SH30071), 100 μg ml^−1^ streptomycin and 100 U ml^−1^ penicillin (Gibco BRL; Catalog number: 10378016) at 37 ℃ in a 5% carbon dioxide (CO_2_) incubator. Cells were transfected after 24 h of incubation, 25 pmol siRNA and 7.5 µl lipofectamine RNAiMAX (Invitrogen, Carlsbad, CA, USA; Catalog number: 13778) were added to each well. The cells were collected 48 h after transfection, and the knockdown efficiency was detected by Western blot (Supplementary Figure 1). Finally, we chose the sequence of siRobo2‐2349 (5’‐CGCGAUUCCAUAUCAAUAATT‐3’) for AAV construct. Then, the viral constructs and vector production and purification of AAV were performed by HanBio Technology Co., Ltd. (Shanghai, China). We constructed the siRobo2 sequence as a short hairpin expression vector in vitro and packaged it as the RNAi interference AAV vector (serotype 9) of Robo2 (AAV‐shRobo2; Contract number: HH20190813YXL‐AAV01). The specific expression of foreign genes in HSC was mediated by constructing an AAV vector carrying a glial fibrillary acidic protein promoter. AAV expressing green fluorescent protein (GFP) was used as control (AAV‐shNC). Then, CCl_4_ and sTREM‐1 treated mice were randomly divided into two groups after 1 week of injection: the CCl_4_+sTREM‐1+AAV‐shNC (*n* = 6) and CCl_4_+sTREM‐1+AAV‐shRobo2 (*n* = 6) groups. The mice of the two groups received tail vein injections of AAV or the corresponding control. After CCl_4_ and sTREM‐1 injection continued for 6 weeks, the mice were sacrificed.

For all procedures, mice were overdose euthanized by intraperitoneal injection of phenobarbital (50 mg kg^−1^); all efforts were made to minimize animal suffering; blood and liver tissues were collected and stored at −80 ℃ or fixed with 4% paraformaldehyde (Shijiazhuang Huawo Kerui Biological Technology Co., Ltd., Hebei, China; Catalog number: 202009) and embedded in paraffin (Shanghai YiYang Instrument Co., Ltd., Shanghai, China; Catalog number: 56–58). All animals received humane care according to the criteria outlined in the ‘Guide for the Care and Use of Laboratory Animals’, and these procedures were approved by the ethics committee of the Second Hospital of Hebei Medical University (approval letter No.: 2020‐AE001). All animal experiments were performed in the experimental animal centre of the Second Hospital of Hebei Medical University.

### 2.2 Isolation and culture of primary HSCs

Primary HSCs were isolated by pronase/collagenase perfusion digestion of the liver in CCl_4_+sTREM‐1+AAV‐shNC and CCl_4_+sTREM‐1+AAV‐shRobo2 groups followed by density gradient centrifugation as previously reported.^21^ Cell viability and purity (>95%) were confirmed by trypan blue staining and autofluorescence. Primary HSCs were cultured in Dulbecco's modified Eagle's medium (DMEM) medium (Gibco BRL; Catalog number: 10566016) supplemented with 10% FBS, 100 μg ml^−1^ streptomycin and 100 U ml^−1^ penicillin. Cells were then collected for Western blot analysis.

### 2.3 Measurements of serum alanine transaminase (ALT) and aspartate transaminase (AST)

The concentrations of serum ALT and AST were determined using an ALT or AST Activity Assay Kit (Nanjing Jiancheng Bioengineering Institute, Jiangsu, China; Catalog number: C009‐2, C0010‐2). The optical density (OD) values were measured by a microplate reader (BioTek, Biotek Winooski, Vermont, USA) at 510 nm.

### 2.4 Histological and immunohistochemistry (IHC) analysis

Paraformaldehyde‐fixed mouse liver tissues were embedded in paraffin, cut into 5 ‐μm thick sections and then stained with haematoxylin‐eosin (HE) (Beijing Legian Biotechnology Co., Ltd., Beijing, China; Catalog number: DH0055) and Sirius red (Beijing Legian Biotechnology Co., Ltd.; Catalog number: DC0041). The paraffin‐embedded liver sections were subjected to IHC following the routine protocols as described.^22^ Anti‐α‐smooth muscle actin (α‐SMA; diluted 1:200; Shanghai Abways Biotechnology Co., Ltd., Shanghai, China; Catalog number: CY5295) and anti‐collagen I (diluted 1:50; Abcam, Cambridge, MA, USA; Catalog number: ab34710) were used for IHC staining. Haematoxylin was for counterstaining. The images were obtained using an upright microscope (Leica, Wetzlar, Germany).

### 2.5 Cell culture

An immortalized human HSC cell line (LX‐2) was acquired from CytoBiotech Co., Ltd. (Guangzhou, China) and well validated by Shanghai VivaCell Biosciences Ltd. (Shanghai, China) (Report No.: VC20190606001). Cells were cultured in DMEM medium supplemented with 10% FBS, 100 μg ml^−1^ streptomycin and 100 U ml^−1^ penicillin. The dose‐dependent and time‐dependent stimulations of sTREM‐1 to cells were tested. The experimental conditions of 100 ng ml^−1^ and 24 h were finally selected for the subsequent experiments. SIS3 (a specific inhibitor of Smad3, 3 μM, 4 h; MedChemExpress, Monmouth Junction, NJ, USA; Catalog number: HY‐13013) and LY294002 [a broad‐spectrum inhibitor of phosphoinositide‐3‐kinase (PI3K), 20 μM, 24 h; MedChemExpress; Catalog number: 154447–36–6] were also used to treat the LX‐2 cells. Lentiviral vectors harbouring Robo2‐specific shRNA (Lv‐shRobo2, Shanghai Genechem Co., Ltd, Shanghai, China; Contract number: GCD0205603) were applied; the transfection efficiency was detected by Western blot (Supplementary Figure 2). Cells were transfected with Lv‐shRobo2 for 24 h and then treated with 100 ng ml^−1^ sTREM‐1 for 24 h.

### 2.6 PolyHis protein pull‐down assay and liquid chromatography‐mass spectrometry (LC‐MS) analysis

Recombinant sTREM‐1 with His tag (His‐tag sTREM‐1) was synthesised by Sino Biological Inc. We performed PolyHis protein pull‐down assay by using a Pierce pull‐down PolyHis protein: Protein Interaction Kit (Thermo Fisher Scientific, Waltham, MA, USA; Catalog number: #21277). Recombinant His‐tag sTREM‐1 protein was used as bait protein, and the cell membrane protein extracted from sTREM‐1‐treated LX‐2 cells was utilized as prey protein. This experiment was divided into three groups: I. Bait +, Prey ‐; II. PolyHis +, Prey +; and III. Bait +, Prey +. The ‘Prey ‐’ in Group I refers to the blank lysis buffer without cell and protein components to remove the system background introduced by Bait and lysis buffer. In brief, we used the high affinity of His and metal cobalt to solidify His on the metal cobalt elution column as bait protein to capture the membrane protein of LX‐2 cells that could bind to sTREM‐1. Then, the elution buffer was analysed by LC‐MS to screen out the membrane receptor proteins that interact with sTREM‐1 and further select the membrane receptors involved in the activation of HSC as potential receptor for sTREM‐1. LC‐MS analysis was finished by Shanghai Bioprofile Technology Co., Ltd. (Shanghai, China). Finally, we examined the expression and localization of sTREM‐1 and its receptor to determine the receptor that interacts with sTREM‐1.

### 2.7 Immunofluorescence (IF)

We used IF to detect whether the recombinant human sTREM‐1 was located on the cell membrane. We treated the LX‐2 cells seeded on cover slips with 100 ng/ml fluorescein isothiocyanate (FITC)‐labelled sTREM‐1 for 24h, and cells were then stained the cell membrane with the red fluorescent probe 1,1'‐dioctadecyl‐3,3,3’,3'‐tetramethylindocarbocyanine perchlorate (DiI) (Beyotime Biotechnology Co., Shanghai, China; Catalog number: C1036) for 15 min. In addition, at 24h after stimulation with FITC‐labelled sTREM‐1, cells were fixed in 4% paraformaldehyde for 30 min and then blocked in 5% bovine serum albumin (BSA) (Solarbio Science & Technology Co., Ltd.; Catalog number: A8020) for 1h at room temperature. The cells were incubated with mouse anti‐Robo2 (diluted 1:50; Cell Signaling Technology, Danvers, MA, USA; Catalog number: #45568) overnight at 4℃. After washing with PBS, cells were incubated with the secondary antibody (Cy3 anti‐mouse IgG; Beyotime Biotechnology Co.; Catalog number: A0521) for 1h at room temperature. Cell slides were incubated with 4’6’‐diamidino‐2‐phenylindole dihydrochloride (DAPI) (Solarbio Science & Technology Co., Ltd.; Catalog number: C0065) to stain nuclei for 5 min. For the paraffin‐embedded liver sections, the sections were incubated with a primary antibody of α‐SMA (diluted 1:200) or collagen I (diluted 1:50) overnight at 4℃. Representative images were obtained using a laser scanning confocal microscope (Olympus, Tokyo, Japan).

### 2.8 Western blot analysis

Primary antibodies used were α‐SMA (diluted 1:1,000), collagen I (diluted 1:200; Affinity Biosciences Ltd., Jiangsu, China; Catalog number: AF7001), p‐Smad2 (diluted 1:100; Affinity Biosciences Ltd.; Catalog number: AF8314), p‐Smad3 (diluted 1:100; Affinity Biosciences Ltd.; Catalog number: AF8315), Smad2/3 (diluted 1:100; Affinity Biosciences Ltd.; Catalog number:AF6367), p‐PI3K (diluted 1:100; Affinity Biosciences Ltd.; Catalog number: AF3242), PI3K (diluted 1:100; Affinity Biosciences Ltd.; Catalog number: AF6242), p‐protein kinase B (p‐Akt) (diluted 1:100; Shanghai Abways Biotechnology Co., Ltd.; Catalog number: CY6569), Akt (diluted 1:200; Shanghai Abways Biotechnology Co., Ltd.; Catalog number: CY5561), Robo2 (diluted 1:50; Santa Cruz Biotechnology, Santa Cruz, CA, USA; Catalog number: sc‐376177) and GAPDH (diluted 1:1,000; Shanghai Abways Biotechnology Co., Ltd.; Catalog number: AB0037). Cells or tissues were lysed using radioimmunoprecipitation assay buffer [1 mM phenylmethylsulfonyl fluoride, 50 mM Tris, 1 mM Ethylenediaminetetraacetic acid, 150mM NaCl, 0.1% sodium dodecyl sulphate (SDS), 1% Triton X‐100, 1% sodium deoxycholate] (Beyotime Biotechnology Co.; Catalog number: P0013) for total protein extraction. For the PolyHis protein pull‐down assay, we extracted the cell membrane protein of LX‐2 cells treated with human His‐tag sTREM‐1 by using a Membrane and Cytosol Protein Extraction Kit (Beyotime Biotechnology Co.; Catalog number: P0033). The proteins were quantified, denatured and electrophorized in SDS‐polyacrylamide gel electrophoresis (Shanghai Epizyme Biotechnology Co., Ltd., Shanghai, China; Catalog number: PG112). After transferred, the polyvinylidene difluoride membranes (Merck Millipore, Billerica, MA, USA; Catalog number: ISEQ00010) were blocked with 5% milk (Solarbio Science & Technology Co., Ltd.; Catalog number: D8340) for 1 h at room temperature and then incubated with the primary antibodies overnight at 4℃. After washed, the membranes were incubated with fluorescence‐conjugated secondary antibodies (LI‐COR Biosciences, Lincoln, NE, USA; Catalog number: 925–32212, 925–32213) for 1 h at room temperature. Finally, the protein bands were visualized utilizing an Odyssey infrared imaging system (LI‐COR Biosciences) and quantified by ImageJ software (National Institutes of Health, Bethesda, Maryland, USA). Protein bands of interest were normalized against GAPDH, and quantitative data were presented as relative density ratios.

### 2.9 Cell viability assay

Cell Counting Kit‐8 (CCK‐8) assays (Shanghai share‐bio Biotechnology CO., Shanghai, China; Catalog number: SB‐cck8m) were used to examine the proliferation of LX‐2 cells. In short, the cells were seeded into 96‐well plates at a density of 1 × 10^4^ cells/well and incubated with sTREM‐1 or PBS for 24 h. Then, the CCK‐8 reagent (10 μl well) was added with serum‐free DMEM medium to a final bulk of 110 μl and incubated for 2 h. The OD values were detected by a microplate reader at 450 nm. For the cell cycle distribution analysis, the LX‐2 cells were seeded into a 6‐well plate and harvested after being incubated with sTREM‐1 for 24 h. Cells were then washed in precooled PBS, fixed in 70% ice‐cold ethanol (Tianjin YongDa Chemical Reagent Co., Ltd., Tianjin, China) overnight and stained with propidium iodide (50 ng ml^−1^) in staining buffer supplemented with RNase A (50 mg ml^−1^) (Beyotime Biotechnology Co.; Catalog number: C1052) at room temperature in the dark for 30 min. Finally, the samples were analysed using a FACSVerse flow cytometer (BD Biosciences, San Jose, CA, USA) in accordance with the manufacturer's guidelines and the data were analysed by ModFit 5.0 software (BD Biosciences).

#### 2.9.1 Wound‐healing assay

LX‐2 cells were seeded into the 6‐well plates (3 × 10^5^ cells/well) and cultured reaching 70% confluence. The monolayer of the confluent cells was scratched by a 200 μl sterile pipette tip. The medium and cell debris were aspirated and replaced with serum‐free DMEM. Photographs were taken after incubation with sTREM‐1 for 0 and 24 h respectively. The distance travelled by cells was measured between the two boundaries of a cellular area.

#### 2.9.2 Statistical analysis

Normally distributed data were expressed as mean ± standard deviation (SD), and non‐normally distributed data were displayed as median with interquartile range. The difference between two groups was analysed by Student's t test. For the difference among diverse groups, one‐way ANOVA was utilized. All statistical analysis was fulfilled by using SPSS software version 19.0 (IBM, Armonk, NY, USA). *P*‐values less than 0.05 were considered statistically significant.

## 3 RESULTS

### 3.1 **sTREM‐1 contributes to hepatic fibrosis in mouse models**.

**FIGURE 1:**
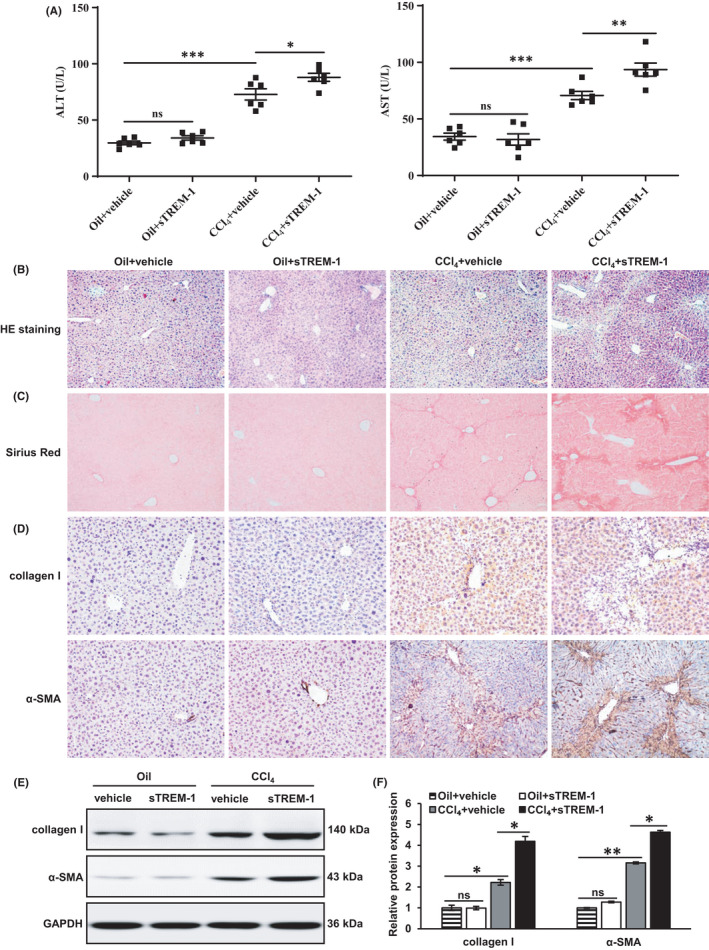
sTREM‐1 promoted the development of liver fibrosis (A) Serum levels of ALT and AST in Oil+vehicle, Oil+sTREM‐1, CCl_4_+vehicle and CCl_4_+sTREM‐1 groups. (B) Liver histology (HE staining, 100X). (C) Liver fibrosis (Sirius Red staining; 100X). (D) Collagen I and α‐SMA expressions (IHC staining, 200X). (E, F) Quantification of collagen I and α‐SMA protein expressions (Western blot). ns: not significant, ^*^ *p* < 0.05, ^**^ *p* < 0.01, ^***^ *p* < 0.001

To clarify the role of sTREM‐1 in the progression of liver fibrosis, we applied the well‐established mouse models of hepatic fibrosis induced by CCl_4_. Tail vein injection of sTREM‐1 resulted in an increase in the serum levels of ALT and AST in CCl_4_‐induced liver fibrosis mice but not in the Oil‐treated mice (Figure 1A). HE staining (Figure 1B) and Sirius Red staining (Figure 1C) showed that there was no abnormality in the morphology of liver tissues in Oil+vehicle and Oil+sTREM‐1 groups, CCl_4_‐treated mice exhibited severe liver injury and fibrosis. Consistently, the expressions of collagen I and α‐SMA (Figure 1D‐F) were significantly increased in the CCl_4_‐treated mice compared with the Oil‐treated mice. Furthermore, the changes in morphology, collagen deposition and the protein expression levels of collagen I and α‐SMA caused by sTREM‐1 were little in the Oil‐treated mice, but sTREM‐1 further exacerbated the degree of liver fibrosis in the CCl_4_‐induced mouse model (Figure 1B‐F). In addition, intraperitoneal injection of sTREM‐1 for a longer period of time, 16 weeks, induced only slight liver damage and collagen deposition compared with the PBS‐treated mice as evaluated by HE (Supplementary Figure 3A) and Sirius Red staining (Supplementary Figure 3B). However, IF showed that there were more hepatic expressions of collagen I (Supplementary Figure 3C) and α‐SMA (Supplementary Figure 3D) in the sTREM‐1‐treated mice than those in the control mice. Taken together, these consequences indicated that sTREM‐1 promoted liver fibrosis and cooperated with CCl_4_ to further aggravate hepatic fibrosis.

### 3.2 sTREM‐1 activates HSCs

**FIGURE 2:**
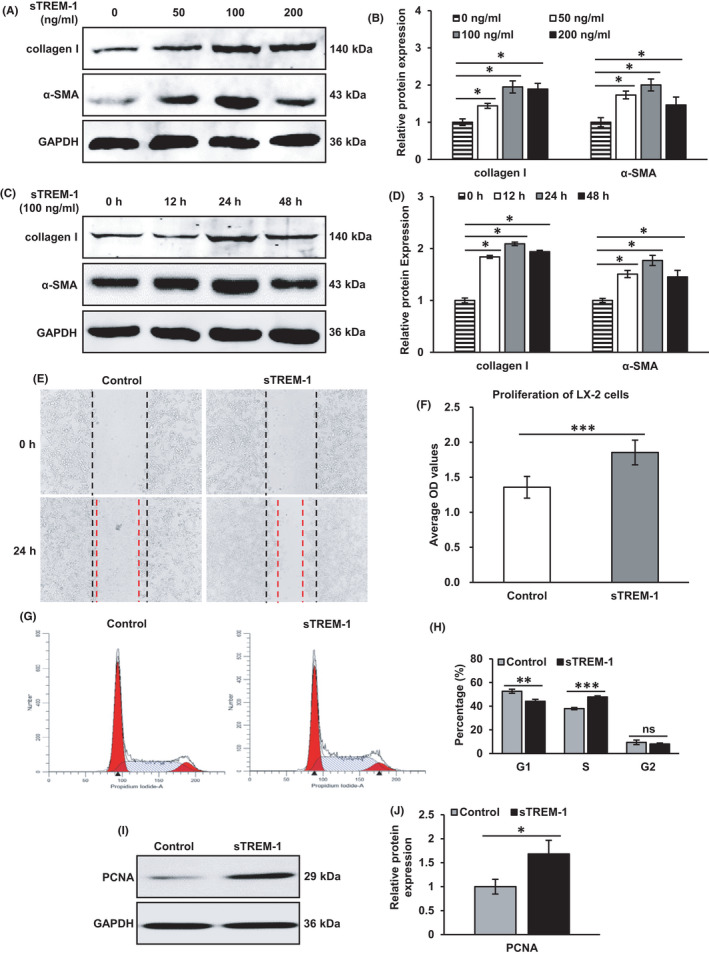
Effects of sTREM‐1 on LX‐2 cells (A‐D) Western blots show that collagen I and α‐SMA expressions are dose‐ and time‐dependence to sTREM‐1 stimulation. (E) Wound‐healing assays showed that sTREM‐1 significantly increased the move distance of HSCs. (F) CCK‐8 assays show that sTREM‐1 significantly increased the HSC viability. (G, H) Flow cytometry shows that sTREM‐1 increased S‐phase and decreased G1‐phase cells. (I, J) Quantification of PCNA protein expressions (Western blot). ns: not significant, ^*^ *p* < 0.05, ^**^ *p* < 0.01, ^***^ *p* < 0.001

We have observed that sTREM‐1 promotes liver fibrosis at the animal level, and the activation of HSC is the core event of liver fibrosis,^23^ so we further explore whether sTREM‐1 could facilitate the activation of HSCs. We stimulated LX‐2 cells with different concentrations of sTREM‐1 (0, 50, 100 and 200 ng ml^−1^) for 24 h, and the α‐SMA and collagen I were significantly increased in a dose‐dependent manner and reached to peak value at 100 ng ml^−1^ (Figure 2A, B). Furthermore, the expressions of α‐SMA and collagen I were significantly increased in a time‐dependent manner and reached to peak value at 24 h (Figure 2C, D). Therefore, we chose to incubate LX‐2 cells with 100 ng ml^−1^ sTREM‐1 for 24 h for subsequent experiments. In the wound‐healing assay as depicted in Figure 2E, the distance moved by wounded cells distinctly increased in the sTREM‐1 groups, and LX‐2 cells transformed into a distinctive elongated phenotype of myofibroblasts after incubation with sTREM‐1 (Supplementary Figure. 4). Furthermore, the viability of sTREM‐1‐treated LX‐2 cells detected by CCK‐8 assays was increased significantly (Figure 2F). Moreover, sTREM‐1 increased S‐phase cells and decreased G1‐phase (Figure 2G, H); and the levels of PCNA were dramatically upregulated in sTREM‐1‐treated cells (Figure 2I, J). The data above indicated that sTREM‐1 enhanced the migration and proliferation of HSC. Therefore, sTREM‐1 facilitated the activation of HSCs.

### 3.3 sTREM‐1 directly bound to cell membrane receptor Robo2

**FIGURE 3:**
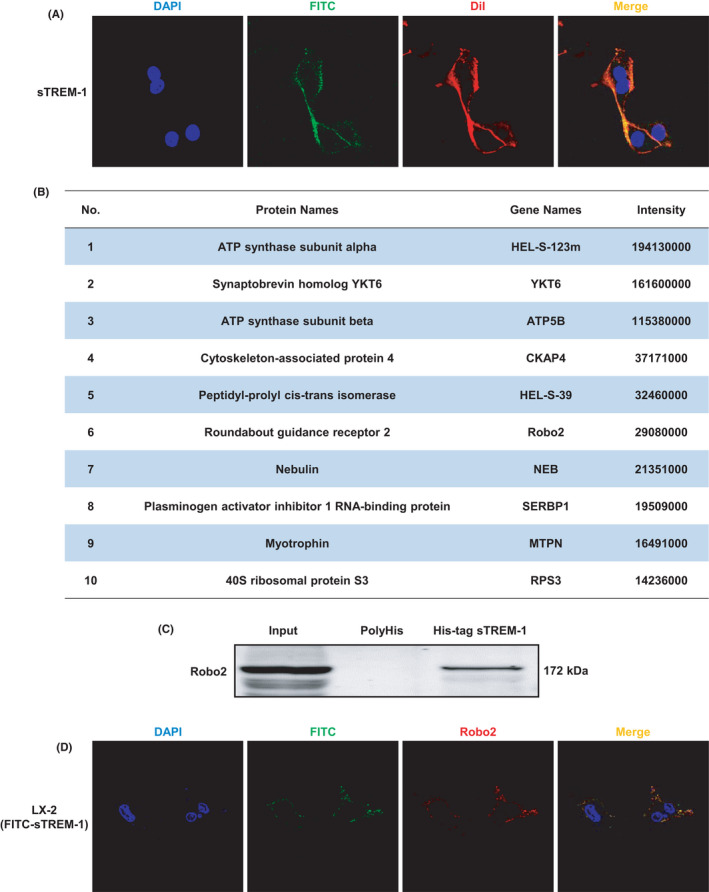
Interaction of sTREM‐1 and Robo2 (A) IF staining showed the attachment of sTREM‐1 on HSC membrane (1,000X). (B) The details of the top 10 membrane proteins in group III (Bait +, Prey +) according to the mass spectrum intensity. (C) Western blot analysis examined the protein expression of Robo2 in Input (cell membrane protein), PolyHis and His‐tag sTREM‐1 groups. (D) The distribution and expression of the FITC label carried by sTREM‐1 and Robo2 were detected by IF (1,000X)

We had proved that sTREM‐1 could induce the activation of HSC and promote liver fibrosis. So, we speculate whether sTREM‐1 may act as a protein ligand that activates downstream signalling pathways by binding to the corresponding membrane receptor. We firstly confirmed that the FITC‐labelled sTREM‐1 was detectable by IF on the DiI‐labelled LX‐2 cell membrane (Figure 3A). To screen out the cell membrane receptor proteins that interact with sTREM‐1, we performed LC‐MS analysis on the elution buffer after pull‐down. We used recombinant His‐tag sTREM‐1 protein as bait protein, and the cell membrane protein extracted from sTREM‐1‐treated LX‐2 cells was utilized as prey protein. In addition, the experiment set up two sets of negative controls (group I: Bait +, Prey – and group II: PolyHis +, Prey +) to exclude non‐specific binding. No cell membrane receptor protein was detected in either group I or II. In Figure 3B, we displayed the information of the top 10 membrane proteins in group III (Bait +, Prey +) according to the mass spectrum intensity; we screened cell membrane receptors related to liver fibrosis among these proteins and found that the sixth protein Robo2 is a membrane receptor. Furthermore, previous study has proved that Robo2 regulates the biological behaviour of HSCs and mediates the development of liver fibrosis,^24^ and we also found that the protein expression levels of Robo2 in liver tissues of mice with liver fibrosis induced by CCl4 were significantly higher than that of the control group (Supplementary Figure. 5). Next, we verified the interaction between sTREM‐1 and Robo2. We firstly used the eluent of LX‐2 cell membrane lysate obtained with PolyHis or His‐sTREM‐1 as bait protein after pull‐down to examine Robo2 by Western blot, and Robo2 was detected in the eluent with His‐sTREM‐1 as bait protein, but no expression in the group with PolyHis as the bait protein (Figure 3C). Secondly, the IF staining revealed the merge of FITC‐labelled sTREM‐1 and Robo2 that co‐localized on the cell membrane (Figure 3D). These results suggest that Robo2 may be a receptor for sTREM‐1 and sTREM‐1 could bind to the cell membrane receptor Robo2 of HSC.

### 3.4 Knockdown of Robo2 inhibited sTREM‐1‐induced HSC activation and the activation of PI3K/Akt and Smad2/3 signalling pathways in vitro

**FIGURE 4:**
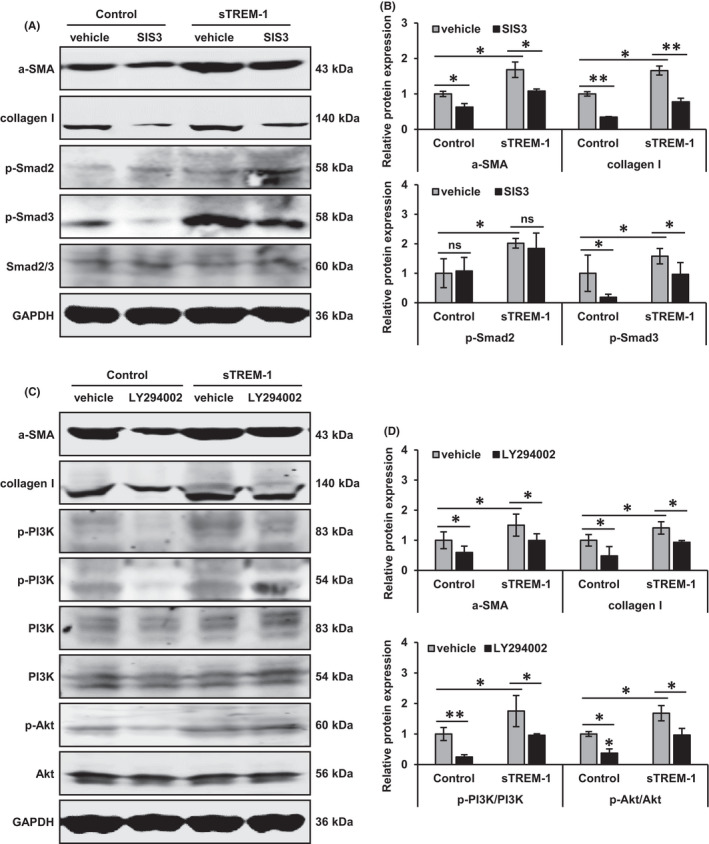
Liver fibrosis‐related protein expressions under Smad2/3, PI3K/Akt inhibitors in LX‐2 cells (A‐D) Western blot analysis showed the protein expressions of α‐SMA, collagen I, p‐Smad2, p‐Smad3, Samd2/3, p‐PI3K, PI3K, p‐Akt and Akt in control, SIS3/LY294002, sTREM‐1 and sTREM‐1+SIS3/LY294002 groups of LX‐2 cells. ns: not significant, ^*^ *p* < 0.05, ^**^ *p* < 0.01

We next further clarify the downstream signalling pathways activated after the binding of sTREM‐1‐Robo2. Previous studies point out that Robo2 regulates both PI3K/Akt and Smad2/3 pathways in liver fibrosis.^24^, ^25^ We, therefore, investigated whether Smad2/3 and PI3K/Akt signalling pathways were involved in sTREM‐1‐induced the activation of HSCs. We found that the stimulation of sTREM‐1 caused significant activation of Smad2/3 and PI3K/Akt signalling pathways, and the suppressions of Smad2/3 by SIS3 and PI3K/Akt by LY294002 significantly attenuated the phosphorylation of Smad3, PI3K and Akt in LX‐2 cells incubated with or without sTREM‐1 (Figure 4A‐D). Furthermore, SIS3 and LY294002 could attenuate the upregulation of collagen I and α‐SMA induced by sTREM‐1 (Figure 4A‐D). These results illustrated that Smad2/3 and PI3K/Akt signalling pathways were involved in the sTREM‐1‐induced fibrogenesis in HSCs.

**FIGURE 5:**
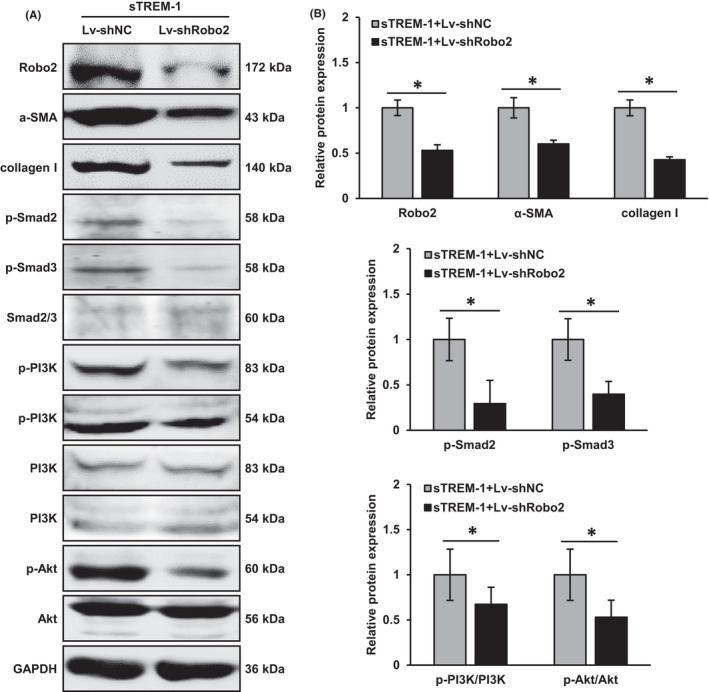
Liver fibrosis‐related protein expressions under Robo2 knockdown in LX‐2 cells (A, B) Western blot analysis showed the protein expressions of Robo2, α‐SMA, collagen I, p‐Smad2, p‐Smad3, Samd2/3, p‐PI3K, PI3K, p‐Akt and Akt in sTREM‐1+Lv‐shNC and sTREM‐1+Lv‐shRobo2 groups of LX‐2 cells. ^*^ *p* < 0.05

When incubated sTREM‐1 with Robo2‐knockdown LX‐2 cells, the expressions of α‐SMA and collagen I were significantly decreased (Figure 5A, B), indicating that Robo2 knockdown suppressed the sTREM‐1‐induced activation of HSC. Furthermore, knockdown of Robo2 suppressed the sTREM‐1‐induced phosphorylation of Smad2, Smad3, PI3K and Akt while the total expression levels of Smad2/3, PI3K and Akt were unchanged (Figure 5A, B). These data revealed that sTREM‐1 activated the PI3K/Akt and Smad2/3 signalling pathways by binding to Robo2, thereby activating HSCs.

### 3.5 Robo2 knockdown suppressed mouse liver fibrosis and inactivated PI3K/Akt and Smad2/3 pathways in vivo

**FIGURE 6:**
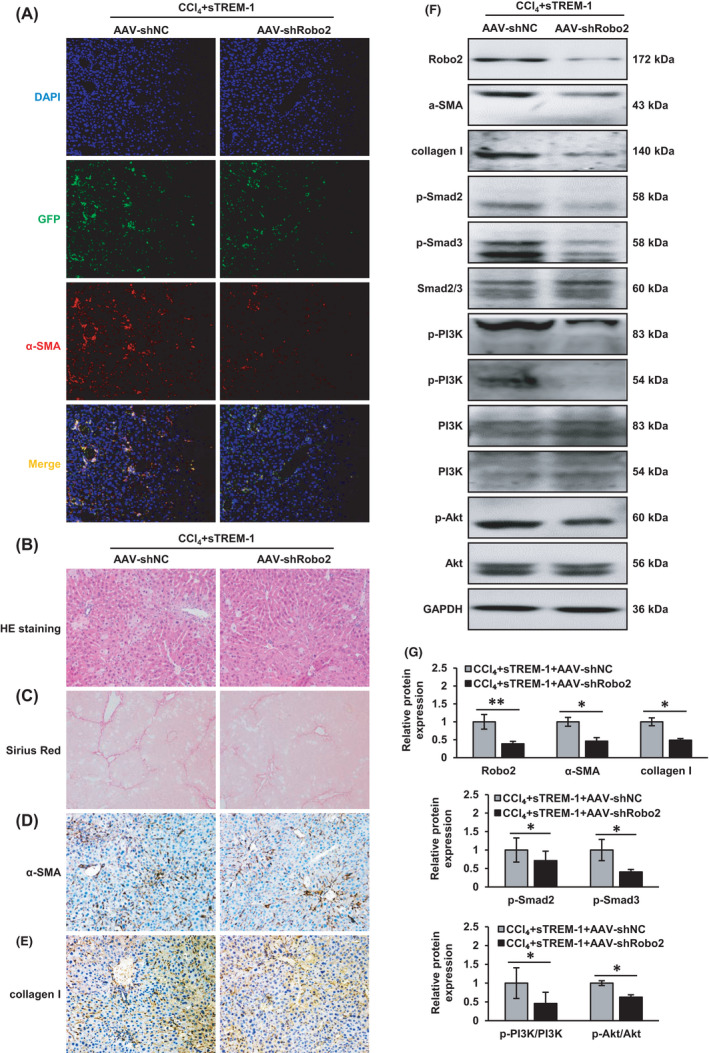
Robo2 knockdown inhibited the development of liver fibrosis and the activation of Smad2/3 and PI3K/Akt signalling pathways in mouse model (A) The distribution and expression of α‐SMA and the GFP carried by the AAV vector were detected in the liver tissue sections of CCl_4_+sTREM‐1+AAV‐shNC and CCl_4_+sTREM‐1+AAV‐shRobo2‐treated mice (200X). (B) Liver histology (HE staining, 200X). (C) Liver fibrosis (Sirius Red staining, 100X). (D, E) α‐SMA and collagen I expressions (IHC staining, 200X). (F, G) Western blot analysis showed the protein expressions of Robo2, α‐SMA, collagen I, p‐Smad2, p‐Smad3, Samd2/3, p‐PI3K, PI3K, p‐Akt and Akt in CCl_4_+sTREM‐1+AAV‐shNC and CCl_4_+sTREM‐1+AAV‐shRobo2 groups of primary HSCs. ^*^ *p* < 0.05, ^**^ *p* < 0.01

Moreover, we further verified the role of membrane receptor Robo2 and its downstream PI3K/Akt and Smad2/3 signalling pathways in sTREM‐1‐related liver fibrosis in vivo. We utilized the IF staining of α‐SMA in liver tissue sections to confirm whether the distribution of AAV‐shRobo2 in the liver was HSC‐specific. IF showed that the GFP carried by the AAV vector was basically consistent with the distribution of α‐SMA, and most of them were combined (Figure 6A), which proved that AAV‐shNC and AAV‐shRobo2 we constructed had the characteristics of specific expression in HSC. All mice here received CCl_4_ and sTREM‐1 intervention, and mice with Robo2 knockdown displayed milder liver damage and collagen deposition than the AAV‐shNC‐treated mice as evaluated by HE (Figure 6B) and Sirius Red staining (Figure 6C). We further isolated primary HSCs from the mice for Western blot analysis. Consistently, lower hepatic expressions of α‐SMA (Figure 6D, F, G) and collagen I (Figure 6E‐G) were observed in the AAV‐shRobo2‐treated mice by IHC and Western blot. The protein levels of p‐Smad2, p‐Smad3, p‐PI3K and p‐Akt were decreased in the CCl_4_+sTREM‐1+AAV‐shRobo2 group while the corresponding total proteins (Smad2/3, PI3K and Akt) were unchanged (Figure 6F, G). These data indicated that reduced combination of sTREM‐1 to Robo2 inhibited the Smad2/3 and PI3K/Akt signalling pathways and the activation of HSC.

## 4 DISCUSSION

So far, scholars have mostly studied the function of TREM‐1 as a receptor, and we pioneered the use of sTREM‐1 as a ligand in liver fibrosis to find receptors that interact with it. To our knowledge, this is the first study on the role of sTREM‐1 in HSC activation and fibrosis. We found that the sTREM‐1‐Robo2‐Smad2/3 and PI3K/Akt pathway plays an essential role in the process of HSC activation and liver fibrosis. sTREM‐1 has synergistic effect with CCl_4_ on liver fibrosis via Robo2‐Smad2/3 and PI3K/Akt pathway, knocking down Robo2 or inhibiting Smad2/3 or PI3K/Akt significantly blocked the sTREM‐1‐induced HSC activation and liver fibrosis.

The inflammatory responses of the liver to variable insults include a series of processes such as HSC activation, phenotypic transformation and collagen secretion.^6^ In the early stage of inflammation, damaged liver cells directly activate HSCs or recruit inflammatory immune cells by releasing damage‐associated molecular patterns.^26^, ^27^ As inflammation progresses, inflammatory immune cells aggregate, release a variety of cytokines such as transforming growth factor‐β1, platelet‐derived growth factor, tumour necrosis factor‐α and interleukin‐6 These cytokines further promote HSCs activation and phenotypic transformation into fibroblasts phenotype with strong proliferation and collagen secretion ability.^26^, ^27^, ^28^, ^29^ Finally, activated HSCs synthesize various extracellular matrix components through autocrine and paracrine.^26^, ^27^, ^29^ Therefore, inflammatory‐related cytokines and their mediated inflammatory response are crucial to the activation of HSC. The sTREM‐1 we studied is a cytokine closely related to inflammation.

Many studies on TREM‐1 have shown that it is associated with various inflammatory diseases. Inhibition of TREM‐1 reduces the inflammatory response during acute gout attacks,^30^ alleviates the neuro‐inflammatory response of Parkinson's disease.^31^ TREM‐1 deficiency attenuates endometritis induced by lipopolysaccharide in mice.^32^HIV or HCV infection stimulates Kuffer cells that upregulates TREM‐1.^33^ In non‐alcoholic fatty liver disease, inhibition of TREM‐1 reduces the inflammation and lipid accumulation.^34^ TREM‐1 is also a poor prognostic factor in HCC patients.^16^ There are still few studies on sTREM‐1 in HCC or other liver diseases, such as sTREM‐1 is significantly increased in the serum of patients with HCC and co‐culture of HSC with HCC significantly increases the secretion of sTREM‐1 from activated HSCs^16^; elevated levels of sTREM‐1 can increase the detection rate of bacterial infections in patients with liver cirrhosis and predict their 90‐day mortality^35^; in addition, sTREM‐1 can also be used as a potential biomarker for the early diagnosis of sepsis in patients with acute‐on‐chronic liver failure.^36^ These studies only focused on the expression of sTREM‐1, but did not discuss its function in liver diseases. Baruah et al. found that sTREM‐1 acts as an antagonist of TREM‐1 by competitively binding the ligands of TREM‐1, inhibiting the TREM‐1 signalling pathway in human neutrophils to exert anti‐inflammatory effects.^37^ Based on this theory, sTREM‐1 should inhibit HSC activation and liver fibrosis. However, our data demonstrated that sTREM‐1 stimulated LX‐2 cells to create more α‐SMA and collagen I in a dose‐ and time‐dependent manner, indicating that sTREM‐1 promoted HSC activation. When we treated the mice with sTREM‐1 for 16 weeks, we definitely found the collagen deposition. The controversy may be due to the target cells. Unlike its effect on neutrophils, sTREM‐1 may directly act on HSCs to activate them in liver fibrosis. Another feature is that sTREM‐1 has no transmembrane domain, and therefore, it acts only on receptors on the cell membrane surface.^17^, ^18^ We screened and verified by pull‐down and LC‐MS analysis that sTREM‐1 acted on Robo2 on the surface of HSC.

Robo protein belongs to a single transmembrane receptor of the Ig superfamily. Four types of Robo proteins have been found in mammals: Robo1, Robo2, Robo3 and Robo4. Its basic structure includes five immunoglobulin‐like domains, three fibronectin type III domains, a single transmembrane segment and an intracellular domain. The intracellular domain does not have any significant catalytic activity,^38^ but there are four short intracellular conserved sequence motifs (CC0, CC1, CC2 and CC3) which are considered to be the binding sites for Robo protein to interact with various intracellular proteins or protein kinases.^39^ Yuen et al.^40^ found that in the mouse models, Robo1 played a regulatory role in the progression of renal fibrosis after ischaemia and obstructive kidney injury. Coincidentally, Chang et al.^25^ further confirmed in the mouse liver fibrosis model induced by CCl_4_ that Robo1 promotes liver injury and fibrosis by activating hepatic stellate cells. They also found that blocking Slit2‐Robo1 signalling inhibits HSCs by inhibiting the Smad2/3 and PI3K/Akt signalling pathways. In addition, Zeng found that Slit2/Robo2 mediates the development of liver fibrosis and regulates the biological behaviour of HSCs by activating PI3K/Akt signalling pathway in a model of thioacetamide‐induced mouse liver fibrosis.^24^ Our research group previously also found that Akt signalling pathway plays an important role in the activation of HSCs and collagen deposition.^2^ These findings indicate that Robo2 and the downstream PI3K/Akt signalling pathways played a definite role in liver fibrosis, and Smad2/3 pathway is also clearly related to liver fibrosis.^25^, ^41^ We selected Smad2/3 and PI3K/Akt signalling pathways as the downstream pathways of sTREM‐1‐Robo2. However, due to the longer coding DNA sequence (4185 bp) of Robo2, it was difficult to achieve its overexpression. The previous study detected the changes in downstream signalling pathways after knocking down Robo1^25^ we, therefore, study the role of Robo2 in liver fibrogenesis via knocking down its expression. sTREM‐1 is a therapeutic target for liver fibrosis, and the advent of short peptide inhibitors^42^, ^43^, ^44^ will greatly increase the possibility of clinical transformation of the sTREM‐1‐Robo2 axis. In addition, sTREM‐1 is also an inflammation‐related biomarker, which will be helpful to explain the role of inflammation in liver fibrosis and further clarify the mechanism of liver fibrosis.

In conclusion, this study innovatively used sTREM‐1 as a novel ligand for membrane receptor Robo2 to explore its effects on HSC activation and liver fibrosis, breaking through the limitation of previous studies that only focused on the functions of TREM‐1 receptor. Our research proved that sTREM‐1 bound to the HSC membrane receptor Robo2 activating the downstream Smad2/3 and PI3K/Akt signalling pathways and clarified its role and mechanism for promoting liver fibrosis. Our study provides a novel insight into the mechanism of liver fibrosis.

## CONFLICTS OF INTEREST

The authors declare that they have no conflict of interest.

## AUTHOR CONTRIBUTIONS

**Ting Liu:** Conceptualization (equal); Formal analysis (equal); Funding acquisition (equal); Methodology (equal); Validation (equal); Writing‐original draft (equal). **Shujia Chen:** Conceptualization (equal); Data curation (equal); Funding acquisition (equal); Methodology (equal); Validation (equal); Writing‐review & editing (equal). **Xiaoli Xie:** Conceptualization (supporting); Methodology (equal); Supervision (supporting); Writing‐review & editing (equal). **Hongqun Liu:** Writing‐review & editing (equal). **Yongjuan Wang:** Conceptualization (supporting); Methodology (equal). **Shengbin Qi:** Methodology (equal). **Linping Shi:** Resources (equal); Validation (equal). **Xue Zhou:** Investigation (equal); Resources (equal). **Jiuna Zhang:** Formal analysis (equal); Methodology (equal). **Shuling Wang:** Methodology (equal). **Yijun Wang:** Investigation (equal); Resources (equal). **Shengxiong Chen:** Investigation (equal); Resources (equal). **Shiying Dou:** Methodology (equal). **Xiaoyu Jiang:** Funding acquisition (equal); Software (equal). **Ruolin Cui:** Data curation (equal); Software (equal). **Huiqing Jiang:** Conceptualization (supporting); Funding acquisition (equal); Supervision (lead); Writing‐review & editing (equal).

## Supporting information

Supplementary Material

## Data Availability

The data supporting the findings of this study are available from the corresponding author upon reasonable request.
