# Supplementary material for: Soluble TREM‐1, as a new ligand for the membrane receptor Robo2, promotes hepatic stellate cells activation and liver fibrosis
Source: J Cell Mol Med. 2021 Nov 9;25(24):11113–27. doi: 10.1111/jcmm.17033 (PMC8650037; doi:10.1111/jcmm.17033)
Supplement: Supplementary file 1 — Supplementary Material [file JCMM-25-11113-s001.pdf]

Supplementary Fig. 1

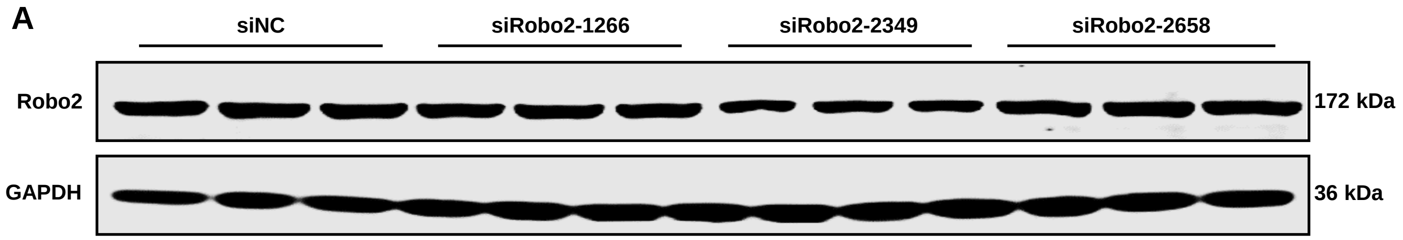

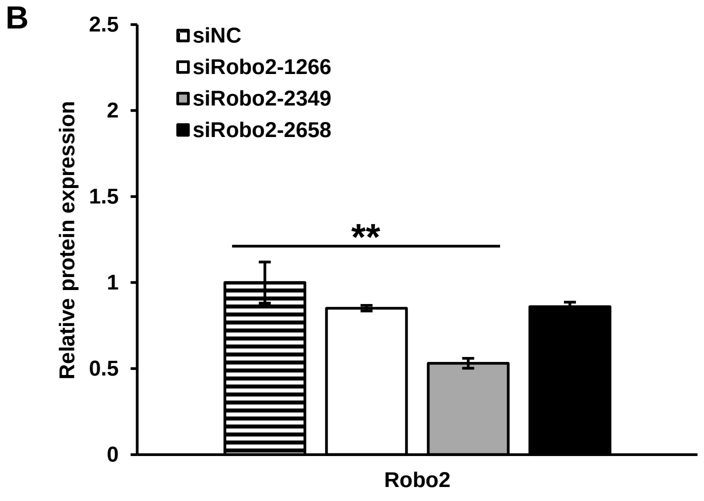

Supplementary Fig. 2

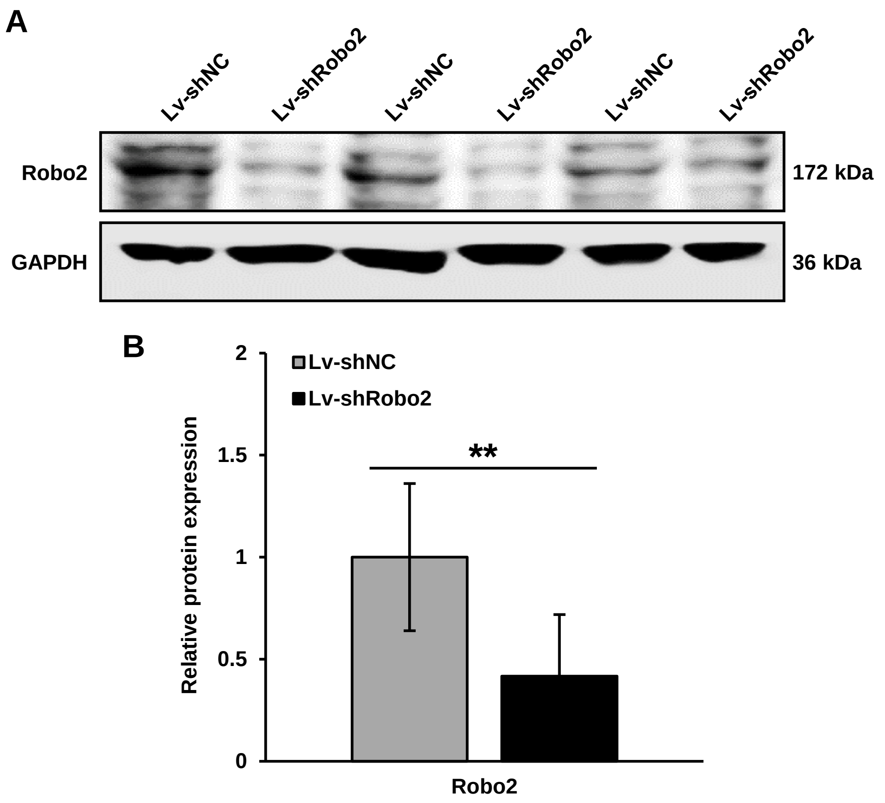

Supplementary Fig. 3

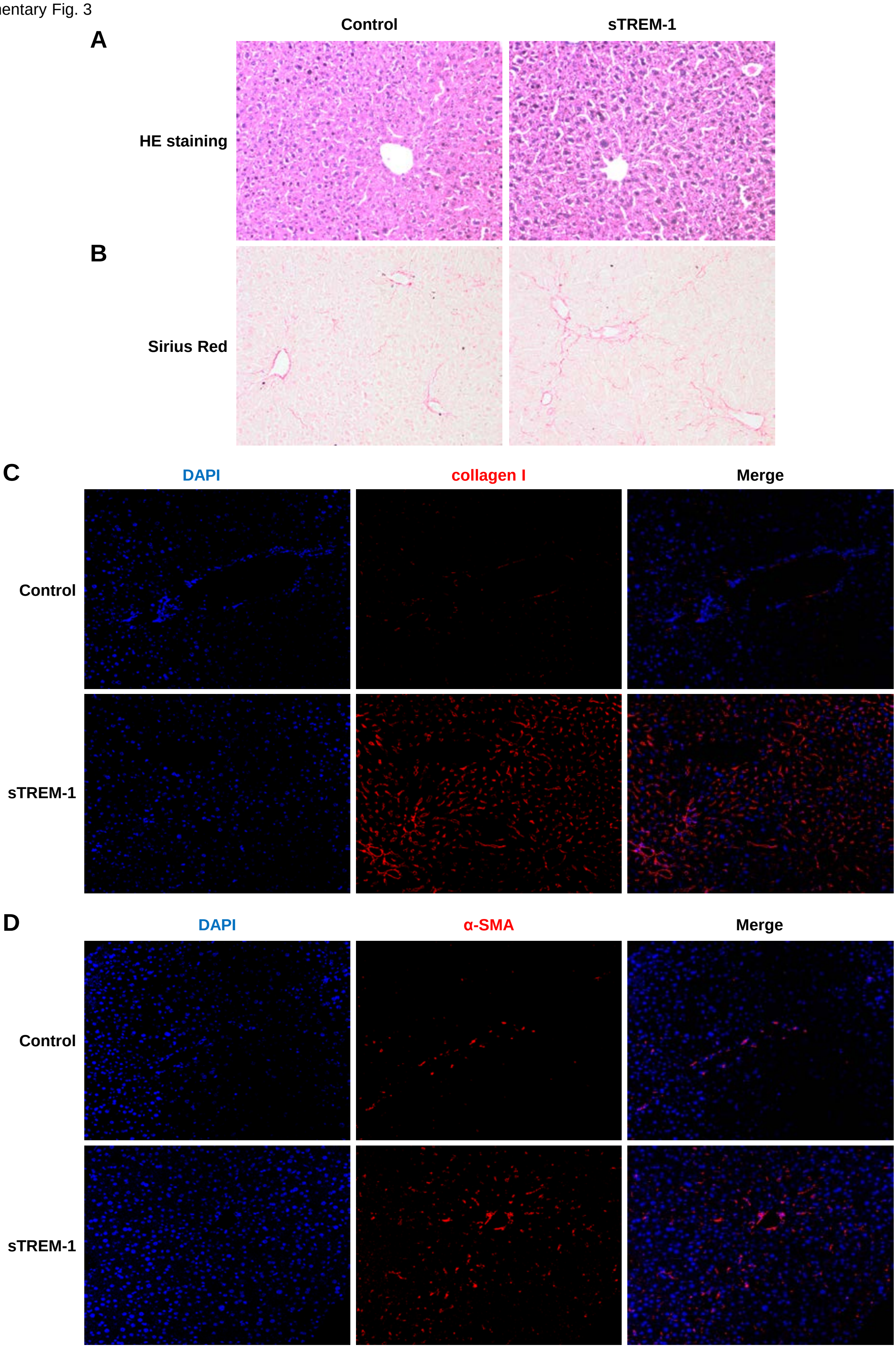

Supplementary Fig. 4

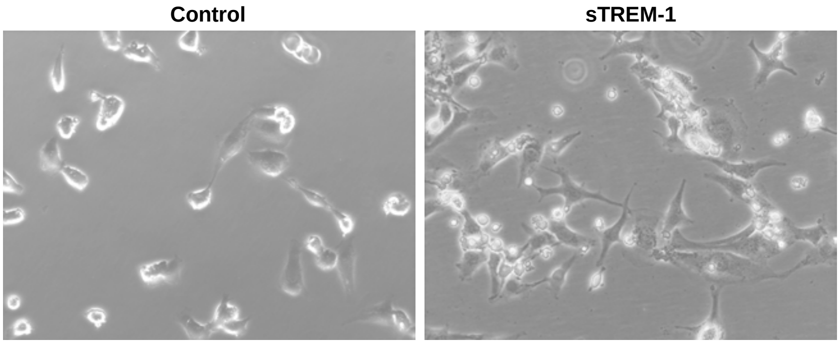

Supplementary Fig. 5

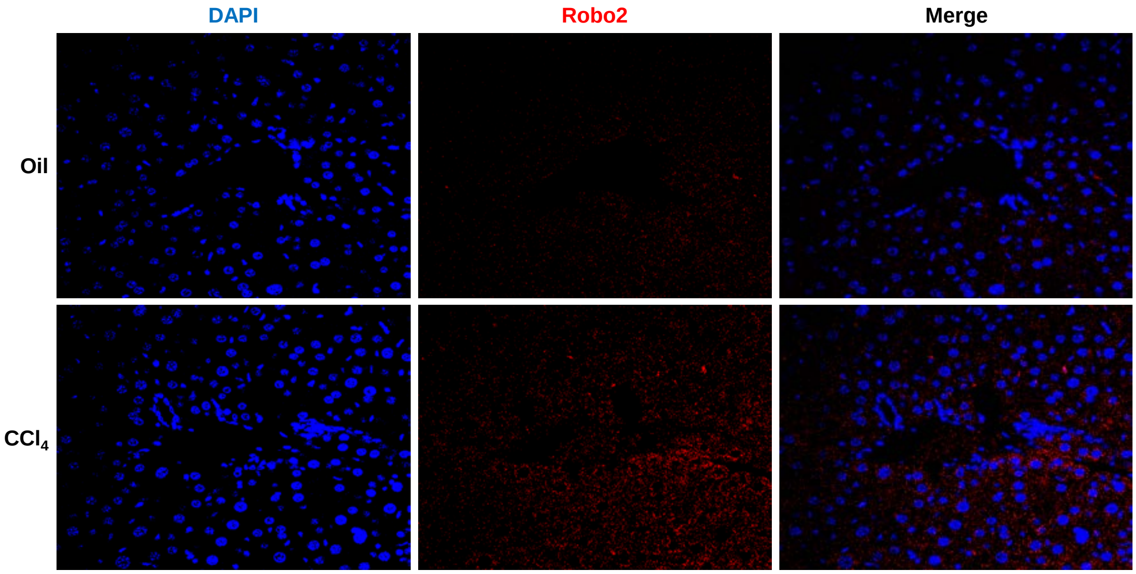

Supplementary Figure Legends

Supplementary Fig. 1 The effective sequence of Robo2 siRNA was screened in CT26 cells (A, B) Western blot analysis showed Robo2 protein expression in siNC, siRobo2-1266, siRobo2-2349, siRobo2-2658 groups of CT26 cells. ** $P < 0.01$.

Supplementary Fig. 2 The transfection efficiency of human Lv-shRobo2 was detected in LX-2 cells (A, B) Western blot analysis showed the Robo2 protein expression in Lv-shNC and Lv-shRobo2 groups of LX-2 cells. ** $P < 0.01$.

Supplementary Fig. 3 sTREM-1 induced the occurance of liver fibrosis (A) Liver histology (HE staining, 200X) was detected in the liver tissue sections of PBS and sTREM-1-treated mice. (B) Liver fibrosis (Sirius Red staining, 100X). (C, D) α-SMA and collagen I expressions (IF staining, 200X).

Supplementary Fig. 4 Morphological changes of LX-2 cells after sTREM-1 incubation LX-2 cells transformed into myofibroblast phenotype after incubation with sTREM-1 for 24 h (200X).

Supplementary Fig. 5 The expression of Robo2 in mouse liver fibrosis The expression levels of Robo2 in CCl_4 group were significantly higher than that of the Oil group (IF staining, 400X).

河北医科大学第二医院科研伦理委员会
Research Ethics Committee of the second hospital of Hebei Medical University

科研伦理委员会伦理审查决议

Approval Letter of Research Ethics Committee

<table>
<tr><td>项目名称
Study Title</td><td colspan="3">一种新的促肝纤维化炎症因子sTREM-1通过与Robo2结合诱导肝星状细胞活化</td></tr>
<tr><td>主要研究者
Principal investigator</td><td>姜慧卿
Huiqing Jiang</td><td>科室
Department</td><td>消化内科</td></tr>
<tr><td>送审日期
Date Submitted</td><td>2020-07-17</td><td>审查决议编号
Approval Letter No.</td><td>2020-AE001</td></tr>
<tr><td>送审资料
Document(s)
Reviewed</td><td colspan="3">实验方案 Experimental scheme
实验动物来源 Sources of laboratory animals
项目负责人资质 Qualification of project leader</td></tr>
<tr><td>项目来源
Issued BY</td><td colspan="3">☐纵向课题 ☐院级课题 ☐横向课题
☐伦理备案 ■论文发表 ☐研究生课题</td></tr>
<tr><td>审查结论
Evaluation</td><td colspan="3">根据该研究的实验设计，经伦理委员会审查，动物实验方法和目的符合人类的道德伦理标准和国际惯例，实验动物的健康、权利等得到充分保护，对实验动物的伤害已控制到最小，动物处置方法合理，能做到尊重动物的生命。
According to the experimental design of the study, after the review of the ethics committee, the method and purpose of animal experiment conform to human moral and ethical standards and international practice. The health and rights of experimental animals have been fully protected, and the harm to experimental animals has been minimized.The method of animal disposal is reasonable and can respect the life of animals.
同意（Approved）在我院实施。
主审委员（签章）
主任委员（签章）
日期：2020 年 9 月 10 日</td></tr>
</table>

地址：河北省石家庄市新华区和平西路 215 号　　邮编：050051　　电话/传真：0311-66002811
Address: No.215 Heping West Road, Xinhua District, Shijiazhuang ,Hebei, 050051,Tel/Fax:0311-66002811

细胞 STR 分型检验报告

Report of Cell Line Identification

客户名称/Applicant：	河北医科大学第二医院
样本编号/ Sample No.：	LX2
待检测细胞系名称/Name of cell line:	LX-2
样本数量及规格/ Sample Spec.：	细胞沉淀 1 个/ Cell precipitation
样本接收日期/ Sample Receive Date：	20190603
报告编号/ Report No：	VC20190606001

1. 测试要求/Service Description

鉴定该细胞样品是否存在交叉污染现象，并与 ATCC 和 DSMZ 数据库比对 DNA 分型数据确认来源。

Detection of human origin intra-species cross-contamination. Database search and analysis to identify cell origin of sample using two recognized repositories (ATCC and DSMZ).

2. 检材处理和检验方法/Method and Procedure

取适量检材用莱枫痕量试剂盒提取 DNA，采用人类 STRtyper-21G 扩增荧光检测试剂盒（Plus）进行复合 PCR 扩增，在 ABI 3730xl 型遗传分析仪上对 STR 位点和性别基因 Amelogenin 进行检测。

Cellular DNA is purified with lifefeng DNA kit. PCR is amplified with SureID 21G Human STR Identification Kit. PCR products are assayed with 3730xl DNA Analyzer (Applied Biosystems).

3. 检验结果/STR Profiles

该细胞株的 STR 位点和 Amelogenin 位点的基因分型结果见附表 1，分型图谱见附图 1。

The STR profiles of the cell line sample are summarized in Table 1 and Figure 1.

4. 检验结论/Result & Analysis

样本编号/Sample No.： LX2

1) 用 GeneMapperID-X 1.4 software(ABI)对各 STR 位点进行基因型分析。该细胞 DNA 扩增后图谱清晰，分型结果良好。
   STR Typing profile is analyzed with GeneMapper ID-X 1.4 software (Applied Biosystems) (Table 1 and Figure 1).
2) 性别基因 Amelogenin：X,Y。
3) 该株细胞 DNA 进行细胞 STR 分型结果显示（如图 1），未出现多等位基因。
   Normal peaks distribution was observed (Figure 1).
4) 该株细胞 DNA 分型在 ATCC & DSMZ 数据库中未找到与其细胞分型完全匹配的细胞。该细胞样本 STR 图谱与 LX-2（Sources: PubMed=24116068）一致，与原描述的细胞相符。
   No 100% matched cell line is found in ATCC & DSMZ data banks. There STR profile was consistent with LX-2 (Sources: PubMed=24116068).

审核人/Reviewed by： 邓昌焕/Changhuan Deng

表 1：样本 LX2 的 STR 位点和 Amelogenin 位点的基因分型结果

Table 1:STR profiles of Sample

	**Sample**	**Source(s): Millipore; PubMed=24116068**
	LX2	LX-2, 100%Match
Marker	Allele	Allele
D19S433	13,15.2	/
D5S818	11,12	11,12
D21S11	28,31	/
D18S51	12	/
D6S1043	11,12	/
AMEL	X,Y	X,Y
D3S1358	13,15	/
D13S317	11,13	11,13
D7S820	11	11
D16S539	13	13
CSF1PO	10,12	10,12
Penta D	12,13	/
D2S441	11,14	/
vWA	17	17
D8S1179	13	/
TPOX	8,9	8,9
Penta E	5,21	/
TH01	9.3	9.3
D12S391	15,17	/
D2S1338	17	/
FGA	21	/

Applied Biosystems
GeneMapper® ID-X 1.4

Project: 2019060501-MR21+MR23+MR29Y

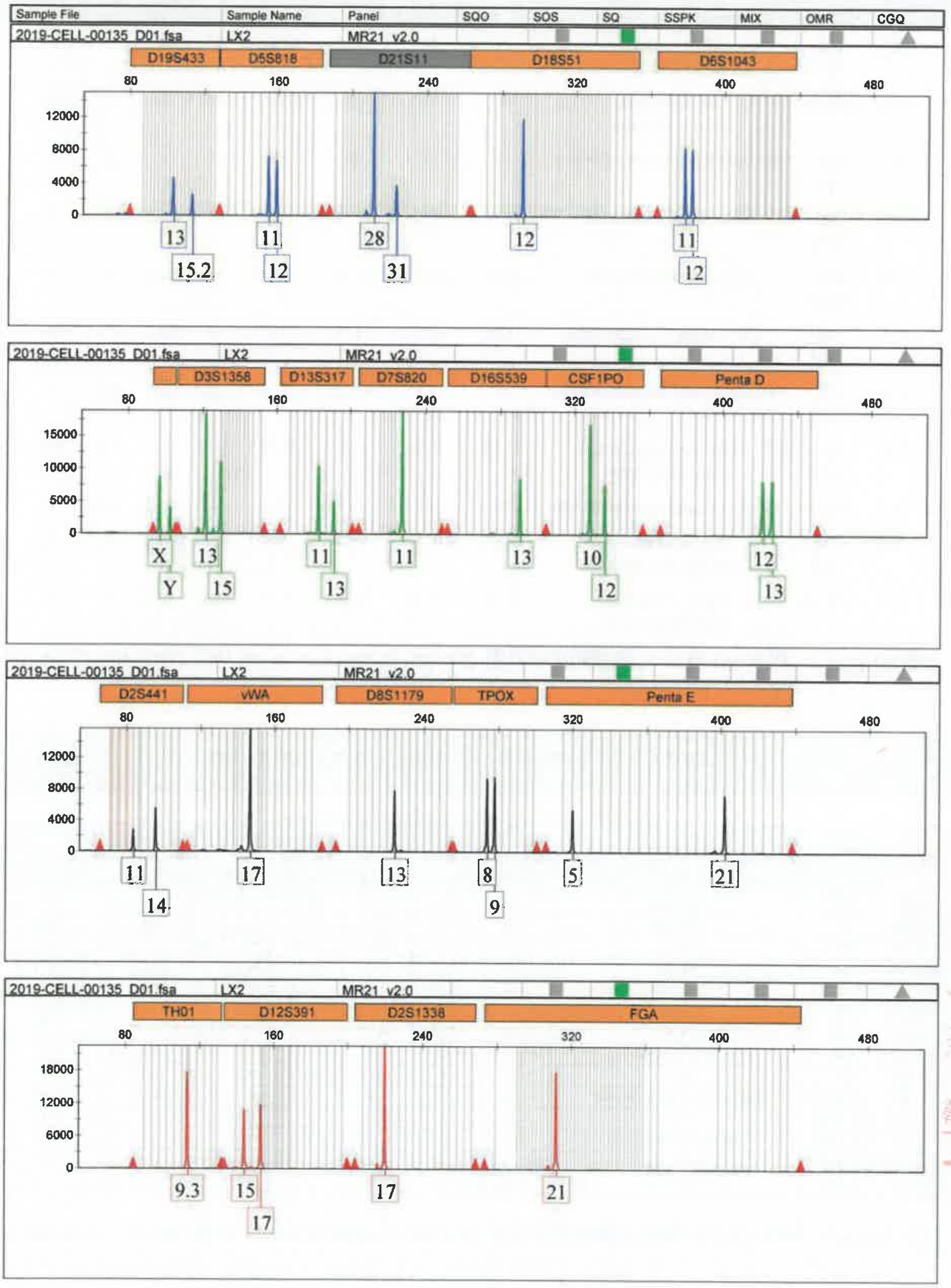

图 1/Figure 1：样本 LX2 的 STR 位点和 Amelogenin 位点的基因分型图（STR profiles of Sample）

Add to Cart	%Match	ATCC® Number	Designation	D5S818	D13S317	D7S820	D16S539	vWA	TH01	AMEL	TPOX	CSF1PO
	82.0	CRL-2331	HCC1599Breast CarcinomaHuman	12	11	10,11	12,13	17	9.3	X	8	12
	78.0	HTB-56	Calu-6Anaplastic CarcinomaHuman	11	11	10	13	17	9	X	8	12
	78.0	CRL-5899	NCI-H1838Lung CarcinomaHuman	12	11	8	13	17	8	X	8	12
	77.0	CRL-9607	HMCBMelanomaHuman	11,12	8,11	11,13	13	17	9,9.3	X	8	10
	77.0	CRL-9446	CHL-1MelanomaHuman	11,12	8,11	11,13	13	17	9,9.3	X	8	10
	77.0	CRL-9451	CHL-2 HUMAN MELANOMA	11,12	8,11	11,13	13	17	9,9.3	X	8	10
	75.0	CRL-5929	NCI-H2171Lung CarcinomaHuman	13	11	11,13	12,13	17	9.3	X,Y	8	10
	73.0	CRL-2235	SNU-182Hepatocelluar CarcinomaHuman	11,12	11,14	11	11,13	17	9	X,Y	8,11	10,12
	73.0	HTB-58	SK-MES-1Lung CarcinomaHuman	11	11	8	13	14	6,9.3	X,Y	8	12
	71.0	HTB-128	MDA-MB-415Breast AdenocarcinomaHuman	11,13	11,13	9,10	13	17	7,9.3	X	8	10,12
	71.0	HTB-80	Capan-2Pancreatic CarcinomaHuman	11,12	11,12	9,11	9,13	17	9.3	X	8	11,12

图 2/Figure 2：样本 LX2 与 ATCC 数据对比分析图（Sample Comparison to the ATCC STR Profile Database）

Result of STR matching analysis by your data.

- DSMZ Profile Database -

A graphical presentation is shown at the bottom of this page.

EV	Cell No.	Cell name	Locus names									Figures
			D5S818	D13S317	D7S820	D16S539	VWA	TH01	AM	TPOX	CSF1PO	
	Query (Your Cell)		*11,12*	*11,13*	*11,11*	*13,13*	*17,17*	*9.3,9.3*	*x,y*	*8,9*	*10,12*	
0.72(26/36)	372	SCLC-21H	11,12	12,12	11,11	12,12	17,17	9.3,9.3	X,Y	8,9	10,10	-
0.72(26/36)	373	SCLC-22H	11,12	12,12	11,11	12,12	17,17	9.3,9.3	X,Y	8,9	10,10	-
0.72(26/36)	763	I83-E95	11,12	11,13	9.3,11	13,13	14,17	6,9.3	X,Y	8,8	10,11	-
0.72(26/36)	764	I83-LCL	11,12	11,13	9,11	13,13	14,17	6,9.3	X,Y	8,8	10,11	-
0.72(26/36)	CRL-2235	SNU-182	11,12	11,14	11,11	11,13	17,17	9,9	X,Y	8,11	10,12	-
0.68(26/38)	245	CAPAN-2	11,12	11,12,13	9,11,11	9,13	16,17	9.3,9.3	X,X	8,8	11,12	-
0.67(24/36)	218	LCL-WEI	11,13	11,12	12,12	13,13	17,17	9.3,9.3	X,Y	8,10	10,11	-
0.67(24/36)	669	UPCI-SCC-154	11,12	9,12	9,10	13,13	17,17	7,7	X,Y	8,9	10,12	-
0.67(24/36)	677	BC-1	11,12	8,8	11,11	12,13	16,20	9.3,9.3	X,Y	8,9	10,11	-
0.67(24/36)	CRL-2172	SW 1990 [SW-1990, SW1990]	12,13	8,12	9,10	13,13	17,17	9.3,9.3	X,X	8,9	10,12	-
0.67(24/36)	CRL-2230	BC-1	11,12	8,8	11,11	12,13	16,20	9.3,9.3	X,Y	8,9	10,11	-
0.67(24/36)	CRL-5969	NCI-BL2171 [BL2171]	12,13	11,12	11,13	12,13	17,17	9.3,9.3	X,Y	8,8	10,13	-
0.67(24/36)	CRL-7276	Hs 402.Sk	11,12	11,13	8,8	13,13	17,17	6,9.3	X,X	8,9	11,11	-
0.67(24/36)	CRL-7581	Hs 848.Sk	11,12	13,14	7,11	11,13	17,20	7,9.3	X,Y	8,9	11,12	-

图 3/ Figure 3：样本 LX2 与 DSMZ 数据对比分析图（Sample Comparison to the DSMZ STR Profile Database）
